# Ti Alloyed *α*-Ga_2_O_3_: Route towards Wide Band Gap Engineering

**DOI:** 10.3390/mi11121128

**Published:** 2020-12-20

**Authors:** Armin Barthel, Joseph Roberts, Mari Napari, Martin Frentrup, Tahmida Huq, András Kovács, Rachel Oliver, Paul Chalker, Timo Sajavaara, Fabien Massabuau

**Affiliations:** 1 Department of Materials Science and Metallurgy, University of Cambridge, Cambridge CB3 0FS, UK; m.p.napari@soton.ac.uk (M.N.); mf562@cam.ac.uk (M.F.); tnh25@cam.ac.uk (T.H.); rao28@cam.ac.uk (R.O.); 2 School of Engineering, The University of Liverpool, Liverpool L69 3GH, UK; j.w.roberts@liverpool.ac.uk (J.R.); pchalker@liverpool.ac.uk (P.C.); 3 Zepler Institute for Photonics and Nanoelectronics, University of Southampton, Southampton SO17 1BJ, UK; 4 Ernst Ruska-Centre for Microscopy and Spectroscopy with Electrons and Peter Grünberg Institute, Forschungszentrum Jülich, 52425 Jülich, Germany; a.kovacs@fz-juelich.de; 5 Department of Physics, University of Jyväskylä, FI-40014 Jyväskylä, Finland; timo.sajavaara@jyu.fi; 6 Department of Physics, SUPA, University of Strathclyde, Glasgow G4 0NG, UK

**Keywords:** gallium oxide, wide band gap semiconductors, solar-blind detection, atomic layer deposition, thin films, alloying, bandgap

## Abstract

The suitability of Ti as a band gap modifier for α-Ga_2_O_3_ was investigated, taking advantage of the isostructural α phases and high band gap difference between Ti_2_O_3_ and Ga_2_O_3_. Films of (Ti,Ga)_2_O_3_ were synthesized by atomic layer deposition on sapphire substrates, and characterized to determine how crystallinity and band gap vary with composition for this alloy. We report the deposition of high quality α-(Ti_x_Ga_1−x_)_2_O_3_ films with x = 3.7%. For greater compositions the crystalline quality of the films degrades rapidly, where the corundum phase is maintained in films up to x = 5.3%, and films containing greater Ti fractions being amorphous. Over the range of achieved corundum phase films, that is 0% ≤ x ≤ 5.3%, the band gap energy varies by ∼270 meV. The ability to maintain a crystalline phase at low fractions of Ti, accompanied by a modification in band gap, shows promising prospects for band gap engineering and the development of wavelength specific solar-blind photodetectors based on α-Ga_2_O_3_.

## 1. Introduction

Alpha phase gallium oxide (α-Ga_2_O_3_) is an ultra-wide band gap semiconductor, with most measurements of its band gap lying between 5.1 eV and 5.3 eV [1,2,3,4,5]. It is of particular interest for applications in solar-blind ultraviolet (UV) photodetectors [2,4,6,7]. Uses for photodetectors that can absorb efficiently in this regime, of wavelengths <285 nm, include water and air purification systems [8], flame detection, UV astronomy, missile defence systems and engine monitoring [4,9].

**Figure 1:**
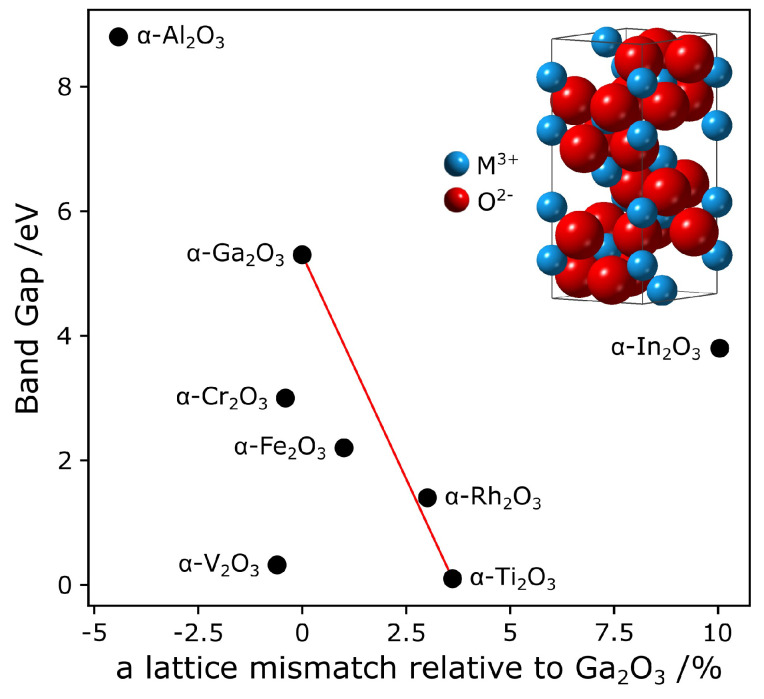
Diagram of the corundum phase semiconducting sesquioxide design space, centered on α-Ga_2_O_3_. The band gaps of the materials [1,27,28,29,30,31,32,33,34,35] are plotted against their ‘a’ lattice parameters relative to α-Ga_2_O_3_ [36,37,38,39,40,41,42]. The red line indicates the alloys of α-Ti_2_O_3_ and α-Ga_2_O_3_, assuming that the band gap varies linearly with the lattice constants. Inset: Rhombohedral, corundum crystal structure of M_2_O_3_ (M: metal).

α-Ga_2_O_3_ is a metastable phase of Ga_2_O_3_, a polymorphic group-III sesquioxide, with commonly reported phases α, β, γ, and ε [10] as well as the more recent κ [11]. Previous research on this material has been mostly focused on the stable, monoclinic β phase [10,12,13], however, due to recent advances in thin film growth techniques, such as mist chemical vapour deposition (mist-CVD) [1,14,15] and atomic layer deposition (ALD) [4,5,16,17], it has become possible to synthesise high quality films of α-Ga_2_O_3_. These films are grown epitaxially on sapphire (α-Al_2_O_3_), which shares its rhombohedral corundum crystal structure [1] (inset Figure 1) with α-Ga_2_O_3_. This has been achieved at temperatures as low as 250 ${}^{{}^{\circ}}$C by ALD [4,5,16] and the material has been successfully integrated into solar-blind UV photodetectors, already showing an advantage over photodetectors based on β-Ga_2_O_3_, by having shorter response times [4].

Apart from sapphire, the corundum crystal structure is also shared by many other semiconducting sesquioxides [18,19,20], as shown in Figure 1, providing great potential for band gap engineering [15,21]. Band gap engineering in the solar-blind region would be pivotal for producing layered device structures and for tuning the application of devices, for example, by producing wavelength-specific biochemical or flame sensors where the material would be tuned to match the peak absorption wavelength of a given micro-organism or molecule [22,23]. Previously, alloying of corundum phase Ga_2_O_3_ with Al_2_O_3_ [24], In_2_O_3_ [25], Cr_2_O_3_ [19], Fe_2_O_3_ [19,20] and Rh_2_O_3_ [26] has been attempted.

The aim of this work is to study the feasibility of using Ti as a band gap modifier for α-Ga_2_O_3_, by characterizing a number of oxide films grown by ALD with different Ti to Ga ratios. α-Ti_2_O_3_ adopts the corundum crystal structure [39,43] with lattice parameters: a = 5.157 Å and c = 13.613 Å [39], giving a relatively small lattice mismatch of about 3.5% with α-Ga_2_O_3_ (a = 4.983 Å and c = 13.433 Å [36]). Its direct band gap of 0.1 eV [29,30] is very small relative to that of α-Ga_2_O_3_, such that a band gap engineering over a few 100 meV (required for wavelength specific solar-blind photodetectors) would be achievable by alloying α-Ga${}_{2}$O${}_{3}$ with only very small amounts of α-Ti${}_{2}$O${}_{3}$. An even wider range of band gaps may be achievable, provided that an alloy of the two sesquioxides exhibits miscibility and crystallinity across a range of Ti:Ga ratios. This may be inhibited if Ti adopts a +4 oxidation state and preferentially forms TiO_2_, which would not have a corundum structure. In β-Ga_2_O_3_, Ti was found to incorporate in the Ga_2_O_3_ host material for low compositions, but led to a phase separated Ga_2_O_3_-TiO_2_ composite at greater compositions [44,45,46]. In the present study it is hoped that the shared corundum structure and low lattice mismatch between α-Ga_2_O_3_ and α-Ti_2_O_3_ would allow to alleviate these issues. Another property of α-Ti_2_O_3_ that is of interest is that it is a p-type semiconductor [30]. Alloying with Ti_2_O_3_ could thus provide a route to achieve p-type conductivity in α-Ga_2_O_3_, as was also demonstrated in an α phase Rh:Ga_2_O_3_ alloy by Kaneko et al. [26].

## 2. Experimental Methods

Ga_2_O_3_ and (Ti,Ga)_2_O_3_ thin films were grown using an Oxford Instruments OpAL plasma enhanced atomic layer deposition (PEALD) reactor. All films were grown on 0.25${}^{{}^{\circ}}$ miscut c-plane sapphire substrates with a temperature of 250 ${}^{{}^{\circ}}$C and chamber wall temperatures set to 150 ${}^{{}^{\circ}}$C. Triethylgallium (TEGa) from Epichem and Titanium(IV) Isopropoxide (TTIP) from Sigma-Aldrich were used as Ga and Ti precursors, respectively. The TEGa and TTIP precursors were held at 30 ${}^{{}^{\circ}}$C and 80 ${}^{{}^{\circ}}$C, respectively, with line temperatures for both precursors set at 90 ${}^{{}^{\circ}}$C and 100 ${}^{{}^{\circ}}$C, increasing in temperature closer to the reaction chamber. One cycle of Ga_2_O_3_ consisted of 0.1 s TEGa with 100 sccm Ar bubbling, 5 s 100 sccm Ar purge, 3 s O_2_ flow stabilisation, 5 s 20 sccm 300 W O_2_ plasma, 5 s 100 sccm Ar purge. One cycle of TiO_x_ consisted of 2 s TTIP with 100 sccm Ar bubbling, 10 s 100 sccm Ar purge, 0.04 s H_2_O, 10 s 100 sccm Ar purge. The chosen growth parameters were adapted from Ref. [47]. Supercycles of Ga_2_O_3_ and TiO_x_ were used to produce Ti alloyed Ga_2_O_3_ films, with cycle ratios (TiO_x_:Ga_2_O_3_) of 0:1, 1:32, 1:19, 1:9, 1:4 and 1:1, which are hereafter referred to as samples 0%Ti, 3%Ti, 5%Ti, 10%Ti, 20%Ti and 50%Ti, respectively. 500 total cycles were used for the 0%Ti film, 429 cycles for the 3%Ti film and 400 cycles for the remaining films. Film thicknesses after growth were measured using a HORIBA Jobin Yvon spectroscopic ellipsometer fitted to a mixed Cauchy β-Ga_2_O_3_/TiO_2_ model and are shown in Table 1. We estimate the film thicknesses obtained using this approach to be within ∼10% accuracy, due to the model itself.

The Ti:Ga ratio of the samples was determined using Rutherford backscattering spectrometry (RBS) with 1.615 MeV He${}^{+}$ incident beam from a 1.7 MV Pelletron accelerator. The samples were tilted to 5${}^{{}^{\circ}}$ and the scattering angle was 165${}^{{}^{\circ}}$. The measured spectra were analysed in the SimNRA program [48] which was used to calculate the Ti:Ga ratios from the respective peak areas in the raw data and weighting them against the corresponding Rutherford scattering cross-sections. Given that the peaks are clearly separated, no fitting was required to analyse these data.

The crystallinity of the samples was assessed by X-ray diffraction (XRD). A PANalytical Empyrean diffractometer was used with a Cu source and a hybrid two-bounce primary monochromator giving Cu Kα_1_ radiation, and either a two-bounce Ge crystal analyser, for 2θ-ω scans, or a PIXcel detector, for reciprocal space maps (RSMs).

High-angle annular dark field scanning transmission electron microscopy (HAADF-STEM) using an aberration-corrected FEI Titan [49] operated at 200 kV was used to observe the sub-surface structure of the samples observed in cross-section. The annular dark field detector semi-angle used was 69.1 mrad. Compositional mapping was obtained using energy dispersive X-ray spectroscopy (EDX) in the same microscope and strain mapping was obtained using geometrical phase analysis [50]. The samples were prepared for imaging using standard mechanical grinding followed by Ar^+^ ion milling at 5 kV and cleaning at 0.1–1 kV.

The surface morphology of the samples was investigated by atomic force microscopy (AFM) in a Bruker Dimension Icon operated in peak force tapping mode. Bruker SCANASYST-AIR tips with a nominal radius of 2 nm were used.

The band gaps of the films were determined from transmittance spectra of the films, measured using a Cary 7000 UV-VIS-NIR spectrometer in the range 200–800 nm. The system was calibrated for 100% and 0% transmittance.

## 3. Results and Discussion

RBS was used for compositional analysis of the films. The main quantity of interest was the ratio of Ti to Ga in the films. RBS spectra were obtained for each film, showing peaks for Ga and Ti (except in the pure Ga_2_O_3_ sample) clearly separated due to the difference in atomic mass, as well as steps associated with Al and O from the sapphire substrate. The compositions x, here defined as x = at.%Ti /(at.%Ti + at.%Ga), attained from the integrated counts of the Ti and Ga peaks in the RBS spectra and corrected for by the respective Rutherford scattering cross sections, are tabulated in Table 1. The film compositions determined by RBS, are in relatively good agreement with those predicted from the ALD cycle ratios, deviating by about 10–30%. Deviations resulting from unequal growth rates of the different species are expected in ALD, with the growth being strongly affected by several factors, including temperature, chemistry of the precursors used, and the growth surface [51,52].

**Figure 2:**
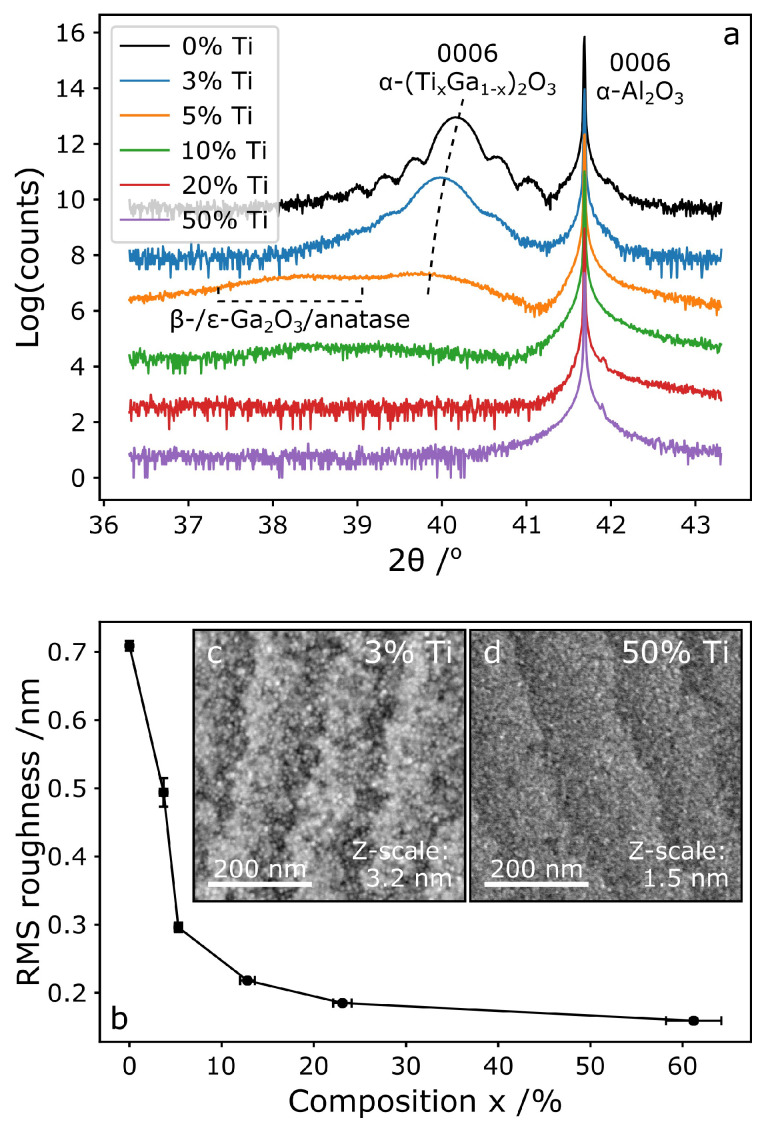
(**a**) 2θ-ω XRD scans of the symmetric 0 0 0 6 reflection of the α phase. (**b**) Variation of RMS surface roughness with Ti concentration. (**c**,**d**) 500 nm size AFM images of the 3%Ti (**c**) and 50%Ti (**d**) samples.

XRD was used to study the crystallinity of the films. The measured 2θ-ω XRD scans (Figure 2a) show the 0 0 0 6 reflection from the sapphire substrate and, for the 3 samples of lowest Ti fraction, also a peak from the film.

The pure α-Ga_2_O_3_ sample (sample 0%Ti) gives the peak of greatest intensity at 2θ = 40.16${}^{{}^{\circ}}$, which is within the range of reported values in epitaxial films (40.05${}^{{}^{\circ}}$–40.25${}^{{}^{\circ}}$ [1,16,36]). Differences in lattice parameter may be due to residual strain from the epitaxial relationship with the substrate. (Annealing the samples at approximately 400 ${}^{{}^{\circ}}$C has been shown to release that residual strain [7]). Interference fringes can be distinguished on the base of the diffraction peak, with a spacing that is representative of the film thickness—here 28 nm, in reasonable agreement with the thickness estimated by ellipsometry (Table 1).

As the concentration of Ti increases, the (Ti,Ga)_2_O_3_ peak becomes broader and weaker, vanishing completely for samples 20%Ti and 50%Ti, which is typical for amorphous materials with very short range order only. For those diffractograms where peaks are observed, that is, for compositions up to 5.3%, the (Ti,Ga)_2_O_3_ peak shifts to smaller angles as the Ti content increases, indicating that the lattice parameter of the crystal increases. This is expected, as the lattice parameters of α-Ti_2_O_3_ are larger and suggests that Ti has been incorporated into the films to form an α-(Ti_x_Ga_1−x_)_2_O_3_ alloy. However, the measured c lattice parameters exceed those expected from the compositions calculated from RBS data, using literature values for lattice parameters and assuming the applicability of Vegard’s Law [53,54]. A likely cause of this is that the α-(Ti_x_Ga_1−x_)_2_O_3_ layers are compressively strained onto the sapphire substrate. However, due to insufficiently strong signals, it was not possible to verify and quantify the strain in the films by conducting a strain analysis from RSMs of symmetric and asymmetric reflections.

Lastly, for sample 5%Ti we note the presence of a second, broad and weak, peak located at a lower angle (∼38${}^{{}^{\circ}}$) than the α-(Ti_x_Ga_1−x_)_2_O_3_ peak (indicated in Figure 2a). Although it is too broad and weak to be uniquely identified, this peak could indicate the presence of one or more additional phases such as β-Ga_2_O_3_, ε-Ga_2_O_3_ or anatase TiO_2_ in this film.

The surface topography of each sample was obtained by AFM and the root mean square (RMS) roughness determined at a 500 nm scan size. The AFM images (Figure 2c,d) are representative of the topographies observed for all samples. The surface was smooth on a nanometer scale, as shown by the RMS roughness given in Table 1 and Figure 2b.

The surface of all samples also bears ledges with a constant spacing, mostly independent of Ti content. These ledges likely arise from the morphology of the sapphire substrates, which, due to having a miscut angle of 0.25${}^{{}^{\circ}}$, also have steps on the surface. These provide two distinct regions: a flat (0 0 0 1) oriented surface and the step. Given preferential growth at one of these sites in ALD, a step pattern with the same spacing would be expected. A topographical scan of a pristine sapphire wafer (not shown here) shows similar step widths as Figure 2c,d.

The surfaces of the low Ti fraction samples (Figure 2c) exhibit small grains of the order of 1–10 nm, presumably due to the termination of α phase columns as was observed in Refs. [5,16]. As the Ti fraction increases (Figure 2d) these features cannot be identified anymore, which may indicate that the films are amorphous, in line with our XRD results. The plot of the RMS roughness vs. Ti fraction (Figure 2b) shows that the roughness decreases quickly with Ti fraction. This decrease in RMS roughness coincides with the disappearance of the small grain features as the Ti fraction increases. This trend (together with the images Figure 2c,d as well as XRD results Figure 2a) implies that the low Ti fraction (x ≤ 5.3%) films are crystalline, whilst the films with high Ti fraction (x ≥ 12.8%) are amorphous.

**Figure 3:**
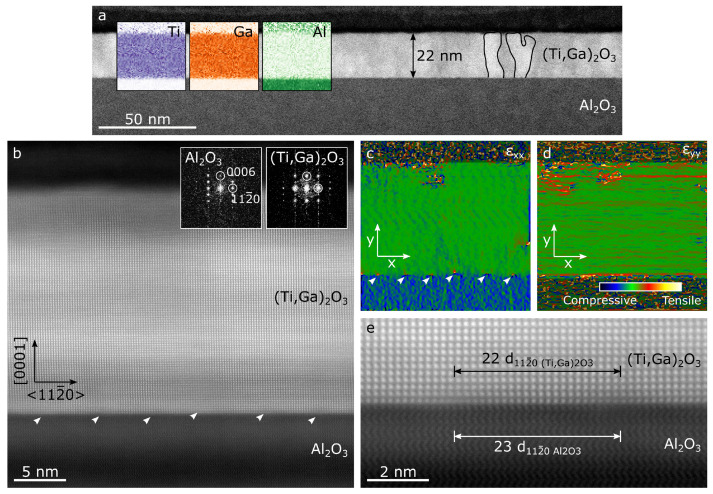
(**a**,**b**) HAADF-STEM images of a cross-section of sample 3%Ti, showing the (Ti,Ga)_2_O_3_ film and its interface with the sapphire substrate. EDS maps are overlaid in (**a**), showing a uniform distribution of Ti and Ga throughout the film. Two columnar regions of crystallinity in the (Ti,Ga)_2_O_3_ film are also outlined in (**a**). In inset of (**b**), the FFTs of the film and substrate confirm the corundum structure. Strain in the film, perpendicular ($g=11\bar{2}0$) (**c**) and parallel (g=0006) (**d**) to the growth direction, was obtained from geometrical phase analysis. Regularly spaced misfit dislocations are indicated by arrows in (**b**,**c**). (**e**) High resolution HAADF-STEM image of film-substrate interface, clearly showing 2 misfit dislocations and their separation. All images are taken along the $\langle 1\bar{1}00\rangle$ zone axis.

STEM was used to analyse a cross section of sample 3%Ti, allowing the interface between substrate and film to be studied as well as giving a local insight to crystallinity, strain and composition across the film. The results are compiled in Figure 3, showing large, vaguely columnar regions of crystallinity in the film (two such columns are outlined in Figure 3a) with, in places, regions of amorphous material. This is in agreement with prior work on ALD grown, pure α-Ga_2_O_3_ [16]. The film thickness measured by HAADF-STEM images (Figure 3a) is consistent with that measured by ellipsometry. We also note that the film is continuous with a fairly uniform thickness, exhibiting only sub-nanometre variation in surface height that is consistent with the low surface roughness obtain from AFM (Figure 2c). The high resolution data and Fast-Fourier transforms (FFT) of the film and substrate regions (Figure 3b) confirm that the film has the corundum structure with the epitaxial relationship with the sapphire substrate being 〈11$\bar{2}$0〉_Al_2_O_3__ ‖ 〈11$\bar{2}$0〉_(Ti_x_Ga_1−x_)_2_O_3__ and [0002]_Al_2_O_3__ ‖ [0002]_(Ti_x_Ga_1−x_)_2_O_3__, in agreement with previous studies [16].

Observation of the interface region reveals the presence of a high density of periodically spaced misfit dislocations, with an average spacing of 4.9 ± 0.4 nm. Apart from these, there are also some dislocations located within the film, approximately 1–3 nm from the interface (not shown here) although these are more rare. The misfit dislocations at the interface can be easily seen in Figure 3b,c (indicated by arrows), the latter showing them as dipoles of tensile and compressive strain. Figure 3e shows two misfit dislocations at greater magnification such that it is possible to count the number of lattice planes between them. They are separated by 23 × d_11$\bar{2}$0 Al_2_O_3__ or 22 × d_11$\bar{2}$0 (Ti_x_Ga_1−x_)_2_O_3__. This indicates that the film is almost fully relaxed (nominally 22 × d_11$\bar{2}$0 Al_2_O_3__ = 5.232 nm, 21 × d_11$\bar{2}$0 Ga_2_O_3__ = 5.232 nm and 21 × d_11$\bar{2}$0 (Ti_x_Ga_1−x_)_2_O_3__ = 5.238 nm assuming x = 3.7%). The strain maps in Figure 3c,d confirm that all the strain relaxation occurs at the interface, and that the strain is otherwise uniform throughout the film. The strain relaxation of the film observed in STEM is in contradiction with the XRD data, which instead suggests that the film is strained. A possible explanation may be that these probe the material at different scales: TEM focuses on a small nanometre-scale region of the sample—typically looking at the scale of an individual column—whereas XRD looks at average/global properties of the film. Another possibility is that the observed imperfect spacing of the misfit dislocations for (Ti_x_Ga_1−x_)_2_O_3_ is unable to fully accommodate the strain.

EDS analysis of the film shows that the distribution of Ti across the film was uniform, with no visible segregation (Figure 3a). Compositional analysis using Cliff-Lorimer method yielded a composition of x ∼ 5%, which is in fair agreement with the RBS data (we note however that EDS quantification is here less reliable than the RBS data, because the sample was imaged on the zone-axis for the α phase and was therefore susceptible to interference from electron channeling).

UV-vis transmittance spectroscopy was conducted to attain the optical band gap of the films. Only transmittance spectra were taken. Ignoring reflected intensity is justified by the fact that the films were transparent and only weakly reflecting to the eye, at normal incidence. Using the assumption of negligible reflectance, Beer-Lambert law could be applied to calculate absorptivity from the transmittance [2,55]. Film transmittances were isolated from the transmittances of the samples by measuring that of a reference pristine sapphire substrate. Absorptivity could then be used to produce Tauc plots using the relation in Equation (1), from which the band gap was determined. A Tauc exponent of n = $\frac{1}{2}$ for a direct energy band gap and allowed transition was used, as was also applied in other works [1,2,3,4,5]:

(1) $$\alpha h\nu \propto {\left(h\nu -E_{g}\right)}^{n}.$$

**Figure 4:**
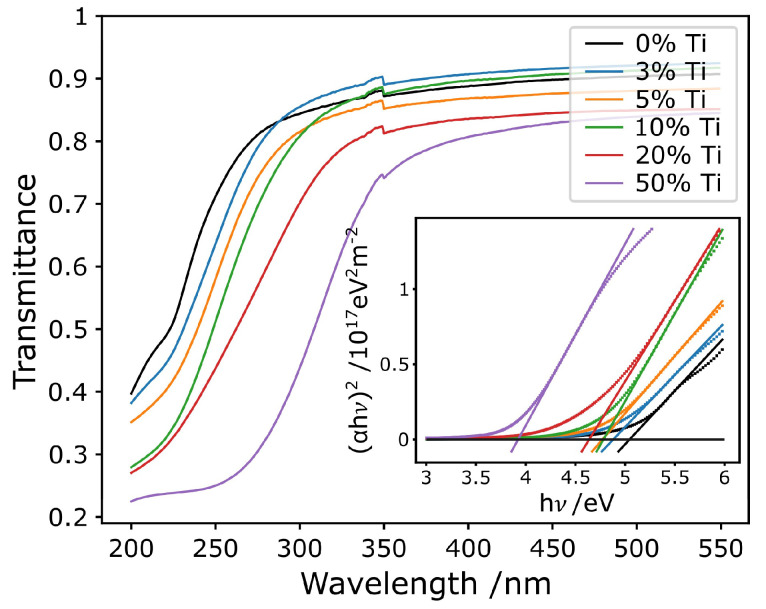
UV-vis transmittance spectra for the (Ti,Ga)_2_O_3_ samples. The inset shows Tauc plots evaluated using the transmittance data for each film. The straight, solid lines are the fits to the linear region of the Tauc plots and their intercepts with the dotted black line (α = 0) gives the band gaps of the respective films.

The collected transmittance spectra for the films are depicted in Figure 4 with an inset of the Tauc plots used to attain the band gap energy. The transmittance spectra clearly show that the absorption edge shifts to longer wavelengths as the Ti concentration of the films increases—as is represented by the band gap energies values listed in Table 1. Above the absorption edge, the transmittance is very high, suggesting that the assumption of little reflection or scattering, when calculating absorptivity is justified (the small discontinuity in the spectrum at ∼350 nm is a systematic instrumental error, due to the source change that occurs in the system at that wavelength). The transmittance at wavelengths longer than the absorption edge seems to decrease with increasing Ti concentration, which could be due to increased scattering or reflection from the films which are not crystalline.

**Figure 5:**
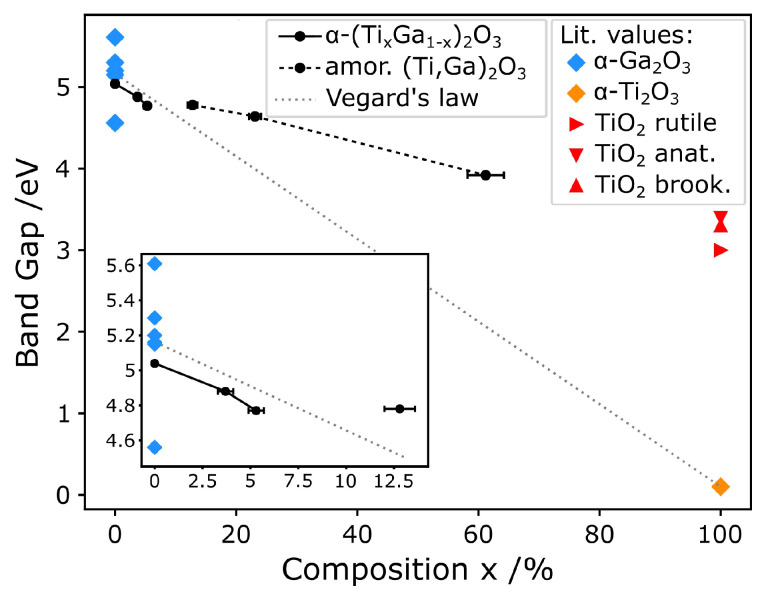
Plot showing the measured variation of band gap energy with the RBS composition alongside literature values of band gaps for pure oxides [1,2,3,4,5,30,56,57,58,59,60,61,62] and a Vegard’s law trend between α-Ga_2_O_3_ and α-Ti_2_O_3_. The inset is an enlargement of the low Ti fraction region. The plot also differentiates between compositions based on whether crystallinity was observed by XRD.

Figure 5 collates the main findings of the study in terms of film composition, crystallinity and optical band gap. The decrease in band gap energy with increasing Ti concentration can be clearly seen. The figure also displays literature values of band gaps for α-Ga_2_O_3_, α-Ti_2_O_3_ and 3 common phases of TiO_2_: rutile [56,57], anatase [58] and brookite [59]. The band gap measured for pure α-Ga_2_O_3_ is well within the range of literature values [1,2,3,4,5,60,61]. The linear trend in band gap with composition predicted by Vegard’s law for α-(Ti_x_Ga_1−x_)_2_O_3_ has been plotted to guide the eye. For the samples with Ti concentration up to 5.3%, that are, the crystalline α-(Ti_x_Ga_1−x_)_2_O_3_ films, the optical band gap seems to follow that trend although this observation should be nuanced as it is based on just 3 data points. Over this range of compositions the band gap varies by ∼270 meV. For the samples with greater Ti concentration, that are, the amorphous films, the band gap follows a different linear trend, with a shallower slope—over this range of composition the band gap varies by ∼1.12 eV.

Given that the solar-blind region covers the range of band gap energies wider than 4.4 eV approximately, our results indicate that α-(Ti_x_Ga_1−x_)_2_O_3_ alloys could be an attractive material for solar-blind photodetector applications. Further improvement in crystal growth could lead to a broader composition range of crystalline films, potentially allowing the production of wavelength-specific photodetectors beyond the solar-blind region.

## 4. Conclusions

Owing to a common crystal structure, low lattice mismatch and wide band gap difference, we investigated the use of Ti as a band gap modifier in α-Ga_2_O_3_. Films of (Ti,Ga)_2_O_3_ with a range of Ti concentrations were synthesized by ALD and the resulting film crystallinity and band gap characterized. The deposition of high quality α-(Ti_x_Ga_1−x_)_2_O_3_ films was achieved for x = 3.7%. The corundum phase was maintained in films of compositions up to x = 5.3%, although the crystalline quality degraded rapidly with increasing Ti fraction. Films with greater Ti content were amorphous. Over the range where corundum phase films were obtained, that is for 0% ≤ x ≤ 5.3%, we report a variation of band gap energy of ∼270 meV. Despite further effort required to increase the crystalline quality of films with greater Ti fraction, this study is a promising proof-of-principle that Ti could be used for band gap engineering of α-Ga_2_O_3_, and opens the path for the fabrication of wavelength-specific optoelectronic devices operating in the UV.

## Figures and Tables

**Table 1:** Summary of film characteristics. Samples are named after the percentage of Ti ALD cycles used for growth. Film thicknesses, obtained by ellipsometry, are estimates, since an appropriate fitting model for α-Ga_2_O_3_, amorphous Ga_2_O_3_ or α-Ti_2_O_3_ was unavailable. Composition, measured by RBS, represents the amount of Ti relative to Ga in the films. RMS roughness and band gaps were determined by AFM and UV-vis transmittance spectroscopy, respectively.

Sample	Thickness/nm	Composition x/%	RMS Roughness/nm	Band Gap/eV
0%Ti	33 ± 3	0	0.71 ± 0.01	∼5.04
3%Ti	22 ± 2	3.7 ± 0.4	0.49 ± 0.02	∼4.88
5%Ti	21 ± 2	5.3 ± 0.4	0.30 ± 0.01	∼4.77
10%Ti	21 ± 2	12.8 ± 0.8	0.22 ± 0.01	∼4.78
20%Ti	21 ± 2	23.1 ± 1.0	0.19 ± 0.01	∼4.63
50%Ti	16 ± 2	61 ± 3	0.16 ± 0.01	∼3.91
